# Supplementary material for: A New Set of Golden-Gate-Based Organelle Marker Plasmids for Colocalization Studies in Plants
Source: Plants (Basel). 2022 Oct 5;11(19):2620. doi: 10.3390/plants11192620 (PMC9572143; doi:10.3390/plants11192620)
Supplement: Supplementary file 1 [file plants-11-02620-s001.zip › plants-1933112-supplementary.pdf]

**Supplemental Materials to**

**"A New Set of Golden Gate-Based Organelle Marker Plasmids for Co-Localization Studies in Plants"**

**by Hagen Stellmach, Robert Hose, Antonia Räde, Sylvestre Marillonnet, and Bettina Hause**

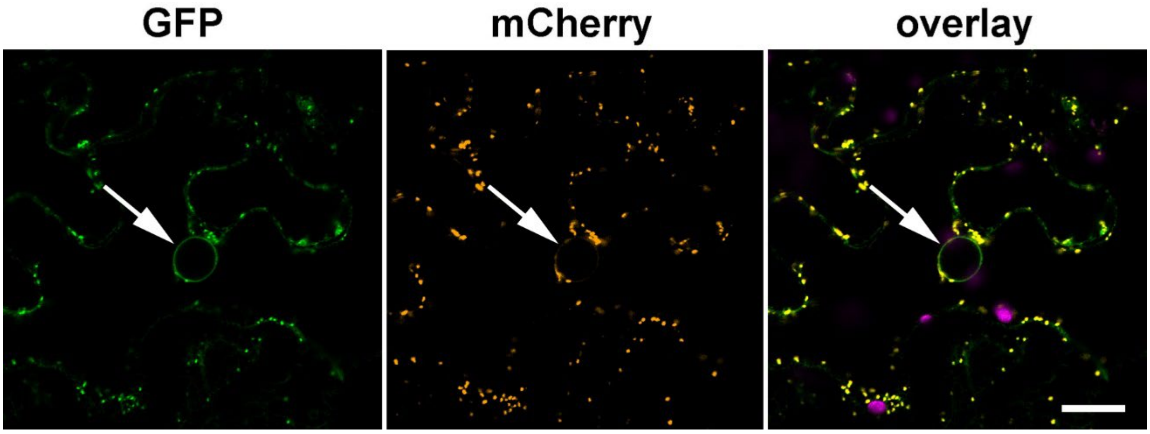


**Figure S1**: Comparison of labeling of Golgi apparatus in epidermal leaf cells of *Nicotiana benthamiana* using co-expression of *p35S::MAN1(1-49)-GFP* (Nelson et al., 2007) and *pNOS::MAN1(1-49)-mCherry*.

Fluorescence of the signal peptide fusion with GFP and mCherry is shown in green and orange, respectively. Note the stronger label of endogenous membranes around the nucleus (arrows) upon expression of the OM under control of the *35S* promoter visible by the green color in the overlay. Bar represents 20 µm for all micrographs.

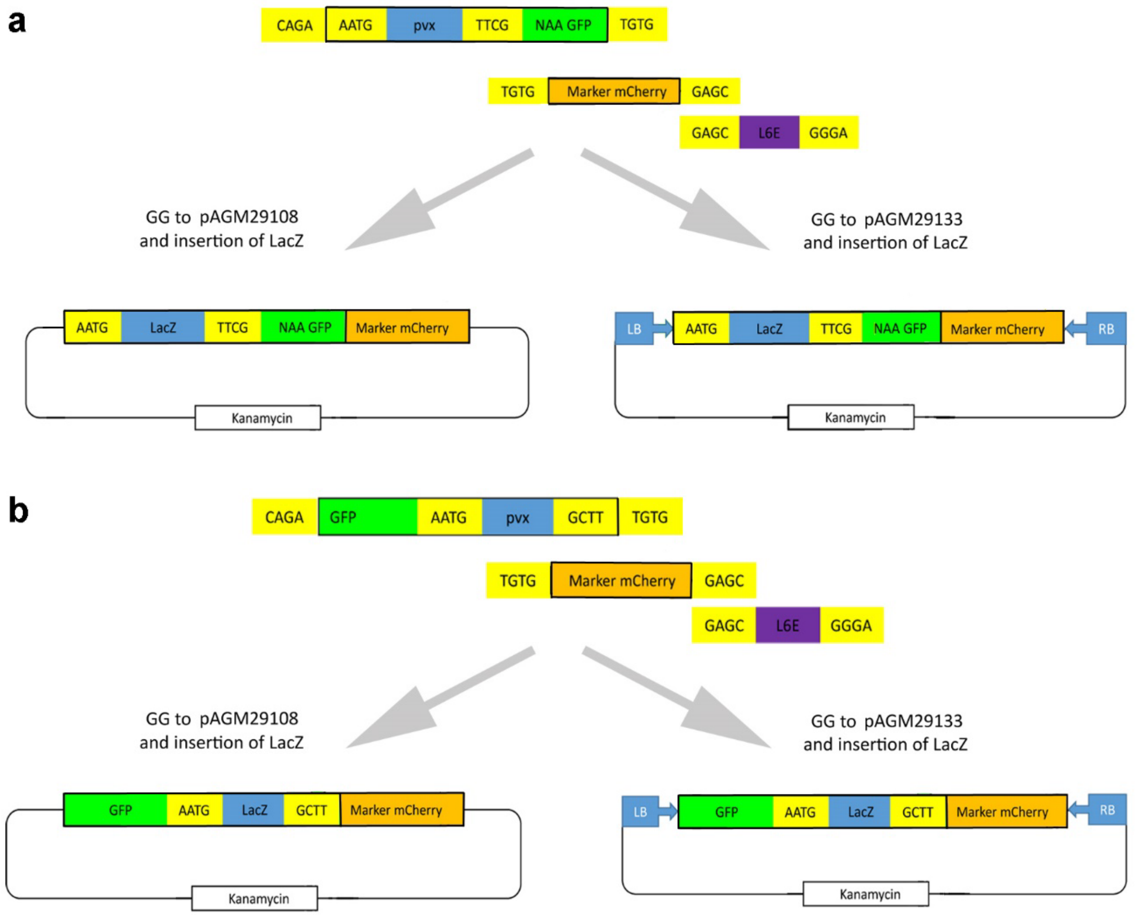


**Figure S2**: Cloning strategy to obtain the final organelle marker vectors for insertion of the coding sequence of the protein of interest (POI).

(a) N-terminal fusion of POI with GFP

(b) C-terminal fusion of POI with GFP

The set created provides for each GFP fusion two vectors per organelle marker, one for PEG-mediated transformation of protoplasts (left) and one for *A. tumefaciens*-mediated transformation of leaves (containing right and left border of the Ti plasmid, right). The cloning site for the coding sequence of the POI allows the respective fusion with GFP and is under control of the *NOS* promoter and *NOS* terminator, whereas the markers are fused to mCherry and are under control of the *NOS* promoter and *35S* terminator. GG = Golden Gate reaction, pvx = potato virus X protein (place holder), L6E = end linker (see {Weber, 2011 #12712}).

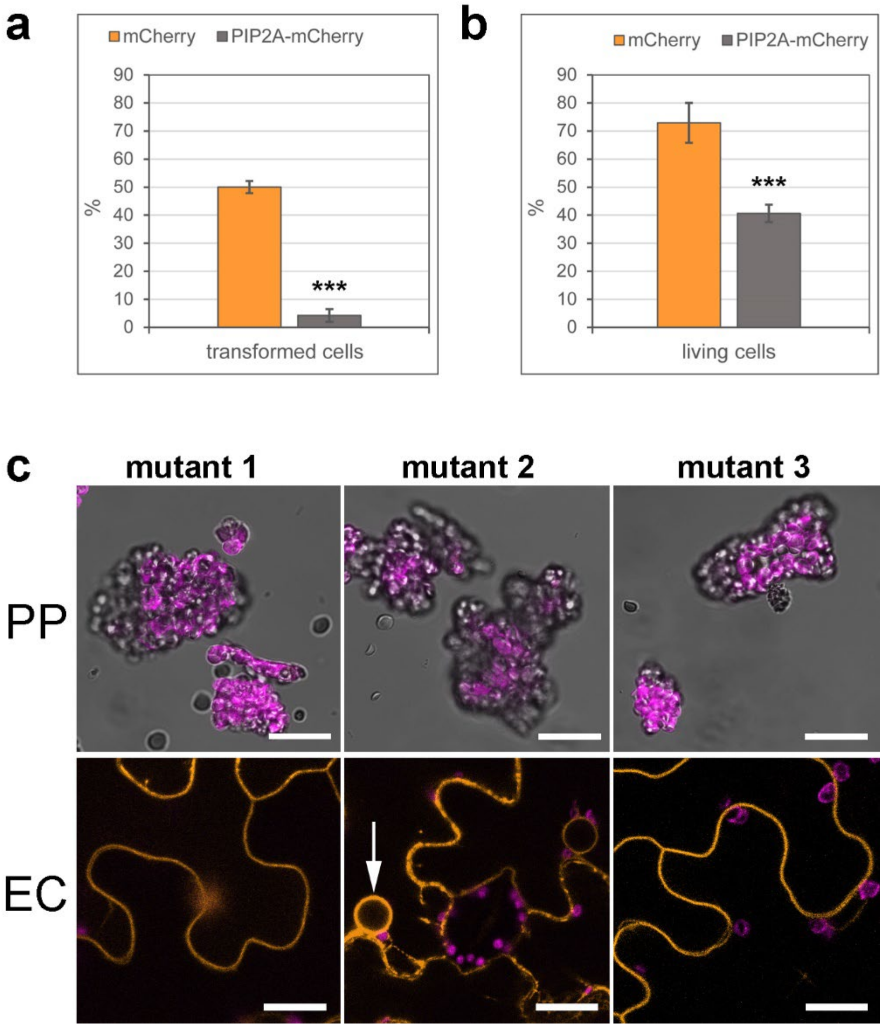


**Figure S3**: Expression of *AtPIP2A* and mutants of it resulted in diminished transformation rates and survival of protoplasts.

a. Transformation rate after protoplast transformation with mCherry (orange) or PIP2A-mCherry (grey).

b. Percentage of living cells after protoplast transformation with mCherry (orange) or *At*PIP2A-mCherry (grey). Vitality of cells was determined using staining with fluorescein-diacetate. Bars in a. and b. represent means and SD (n=3). *** depicts statistical significant difference with P<0.005 according to Student's *t*-test.

c. Subcellular localization of mutated versions of *At*PIP2A in mesophyll protoplasts and epidermal cells of *N. benthamiana*. mutant 1: K3A, mutant 2: A109C (first NPA mutated to NPC), mutant 3: A230C (second NPA mutated to NPC). Note the collapse of mesophyll protoplasts after transformation with the constructs encoding mutated versions of *At*PIP2A. For all three variants, the label appears mainly at the plasma membrane, but also in the endomembrane system in epidermal leaf cells after transformation of mutants variant 2 (arrow). To visualize the protoplasts, the bright field image was added. Fluorescence of the protein fusion with mCherry is shown in orange, whereas the autofluorescence of chloroplasts is depicted in magenta. PP – protoplast, EC – epidermal cell. Bars represent 20 µm in all micrographs.

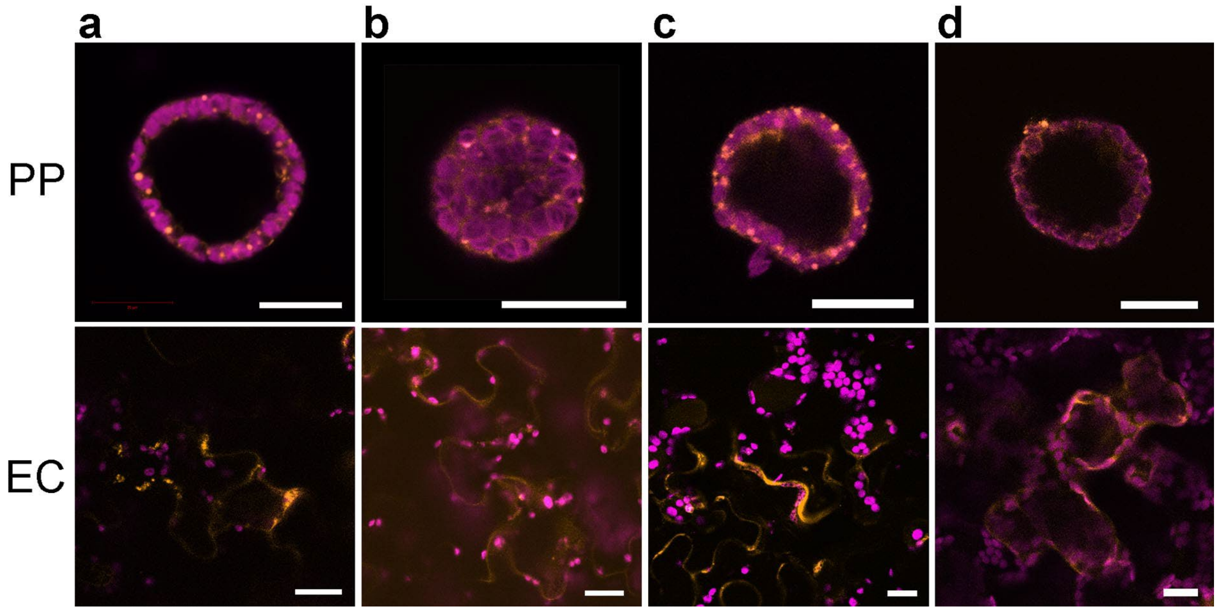


**Figure S4**: Subcellular localization of proteins assumed to be located at plasma membrane (a, b) and tonoplast (c, d).

(a) AHA2-mCherry. (b) NPSN12-mCherry. (c) VAMP711-mCherry. (d) CHNB-mCherry.

Transformation with all constructs led to label appearing within the cytosol or small vesicles, both in mesophyll protoplasts and epidermal cells. In all cases, no clear label of plasma membrane (a, b) or tonoplast (c, d) was visible. Fluorescence of the protein fusion with mCherry is shown in orange, whereas the autofluorescence of chloroplasts is depicted in magenta. PP – protoplast, EC – epidermal cell. Bars represent 20 μm in all micrographs.

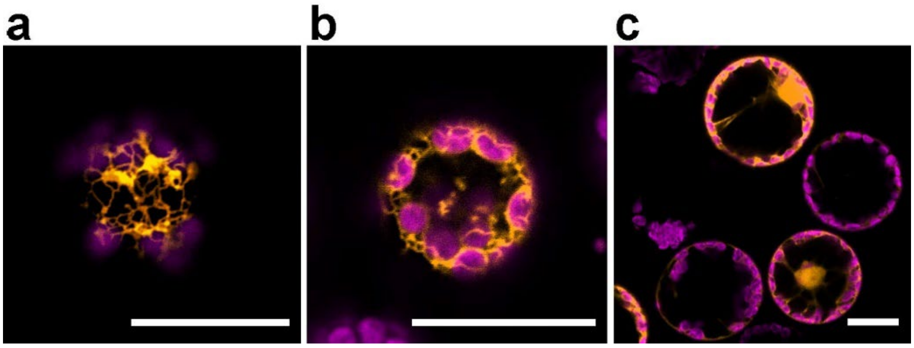


**Figure S5**: Subcellular localization of proteins located within endoplasmic reticulum.

(a) WAK2(1-30)-mCherry-KDEL. In addition to the picture shown in Figure 3, another optical section of the mesophyll protoplast is shown to visualize the tubular network of the labelled ER.

(b) WAK2(1-30)-mCherry-HDEL. The label appears clearly within the ER network.

(c) mCherry-HDEL. Note that the label is not restricted to the ER, but appears within the cytosol and nucleus.

Fluorescence of the protein/signal peptide fusion with mCherry is shown in orange, whereas the autofluorescence of chloroplasts is depicted in magenta. Bars represent 20 µm in all micrographs.

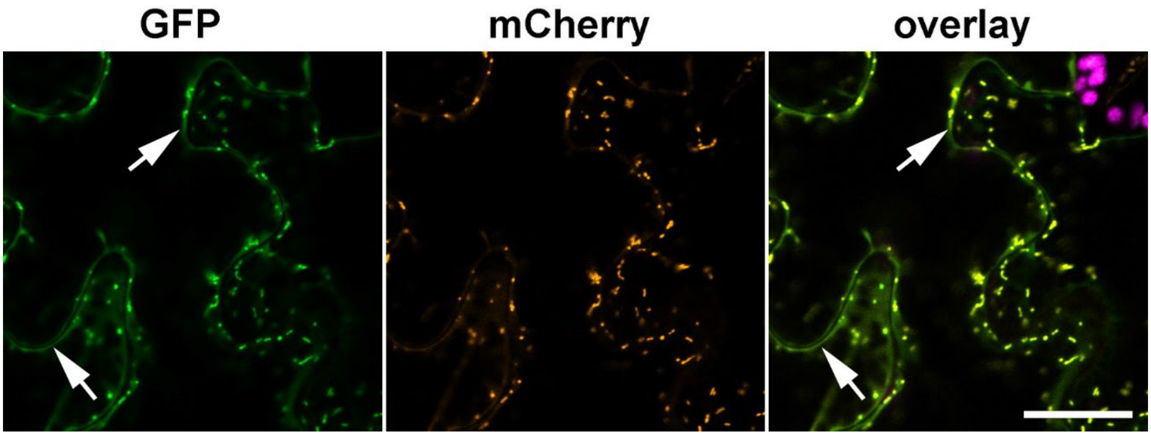


**Figure S6**: Comparison of labeling of mitochondria in epidermal leaf cells of *Nicotiana benthamiana* with COX IV(1-29)-GFP and mtRi(1-100)-mCherry using co-expression of the respective constructs.

Fluorescence of the transit peptide fusion with GFP and mCherry is shown in green and orange, respectively. Note the stronger label of cytoplasm upon expression of *COX IV(1-29)-GFP* (arrows). In the overlay, autofluorescence of chlorophyll is included and depicted in magenta. Bar represents 20 µm for all micrographs.

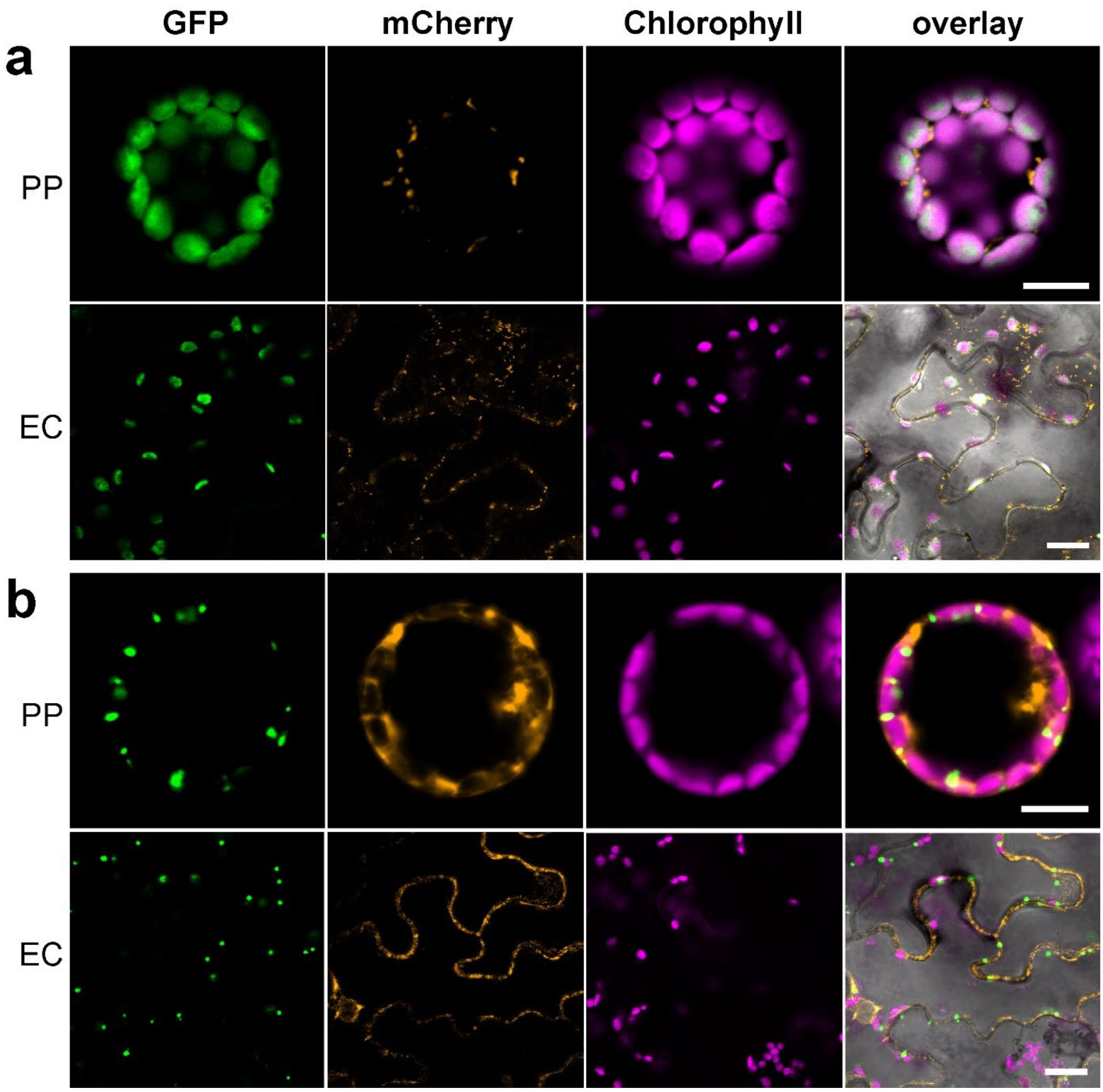


**Figure S7**: Non-co-localization of *At*AOC2 (a) and *At*OPR3 (b) with the respective markers.

(a) The coding sequence of *AtAOC2* was cloned into the final OM (pAGH1091 and pAGH1109), which led to C-terminal fusion of *At*AOC2 with GFP and contained the marker for mitochondria (mtRi[1-100]-mCherry).

(b) The coding sequence of *AtOPR3* was cloned into the final OM (pAGH1130 and pAGH1136), which led to N-terminal fusion of *At*OPR3 with GFP and contained the marker for ER (WAK[1-30]-mCherry-KDEL).

Fluorescence of the target protein fused to GFP is shown in green, whereas the organelle marker (mCherry) is shown in orange. The autofluorescence of chloroplasts is depicted in magenta. The overlay shows all three channels, for epidermal cells the bright field image was included. Note that the GFP fluorescence does not overlap with the fluorescence of the respective organelle marker.

PP – protoplast, EC – epidermal cell. Bars represent 10 µm for protoplasts and 20 µm for epidermal cells.

**Table S1**: Primers used for cloning of marker candidates (overhangs for Golden Gate cloning in red)

| **Candidate gene** | forward primer 5´--> 3´ | reverse primer 5´--> 3´ |
|---|---|---|
| *Plasma membrane intrinsic protein 2 A* | TTGAAGACAATACTGGACAGCCCAGATCAA | TTAGAAGACAACATTACGTTGGCAGCACTTC |
| *H(+)-ATPase 2* | TTGAAGACAACCATGTCGAGTCTCGAAGATAT | TTGAAGACAACCCACAGTGTAGTGACTGGGAG |
| *Novel plant snare 12* | TTGAAGACAACCATGGCGTCTGAATTGCCGATGAG | TTGAAGACAACATTCCTTCTCTGAAGTACAAGAGCTTTCTCGA |
| *Chitinase B* | TTGAAGACAACCATGAGGCTTAGAGAATTCAC | TTGAAGACAACACCCATAGTATCGACTAAAAGTC |
| *V-ATPase* ε-subunit (E1) | ATGAACGACGGAGATGTATC | TTGAAGACAACAGGCAGTAACTTGGCCGAACA |
| *Vesicle associated membrane protein 711* | TTGAAGACAACCATGGCGATTCTGTACGCCCTCGTGGCTC | TTGAAGACAACATTCCAATGCAAGATGGTAGAGTAGGTCCGTGGCAGAG |
| *Cation diffusion facilitator 1* | TTGAAGACAACCATGGAGTCCTCAAGTCCCCACCATAG | TTGAAGACAACATTCCGCGCTCGATTTGTATCGTGACATG |
| *Histone H2A W6* | TTGAAGACAATACTATGGAATCCACCGGAAAAGTG | TTGAAGACAACATTCCAGCTTTCTTTGGAGACTTGACTG |
| *Wall-associated kinase2* (aa 1-30) | TTGAAGACAATACTATGAAGGTACAGGAGGGTTTG | TTGAAGACAAAAGCTTACAGCTCGTCATGAGATCTCTT |
| *mCherry-HDEL* | TTGAAGACAAAATGAAGGTACAGGAGGGTTTG | TTGAAGACAAAAGCTTACAGCTCGTCATGAGATCTCTT |
| *mCherry-KDEL* | TTGAAGACAAAATGAAGGTACAGGAGGGTTTG | TTGAAGACAAAAGCTTACAGCTCGTCCTTAGATCTCTT |
| *α-1,2-Mannosidase* (aa 1-49) | TTGAAGACAATACTGGACAGCCCAGATCT | TTGAAGACAACATTCCGGGACCGGTCCTCG |
| *mCherry-SKL* (PTS1) | TTGAAGACAAAATGGTGAGCAAGGGCGAGGAG | TTGAAGACAAAAGCTTACAGCTTCGATCTCTTGTA |
| *Ribulose-1,5-bisphosphate-carboxylase/-oxygenase* (aa 1-79) | TTGAAGACAATACTCAGATCAACTAGTCTTAAGTCCGGAG | TTAGAAGACAACATTGACAAATCAGGAAGGTATGAGAGAGTCTC |
| *Mitochondrial Rieske protein* (aa 1-100) | TTGAAGACAATACTATGCTTCGAGTAGCAGGTAG | TTGAAGACAACATTCCGCTAGGATCTCCAGGTGGAT |

**Table S2**: Primers used for creating mutants of Plasma membrane intrinsic protein 2 A (PIP2A)

| **Mutant version** | forward primer 5´--> 3´ | reverse primer 5´--> 3´ |
|---|---|---|
| Mutation 1: K3A | TAGAATGGCAGCGGATGTGGAAGC[illegible] | GAGCTAGCTCCGGACTTA |
| Mutation 2: A109C | CATTAACCCATGCGTGACATTTGGGCTATTC | TGACCACCAGAGATACCG |
| Mutation 3: A230C | AATTAACCCGTGCAGGAGTTTCGGAGCTGC | CCGGTTCCGGTAATGGGA |
